# Characterization of 500 Chinese patients with cervical esophageal cancer by clinicopathological and treatment outcomes

**DOI:** 10.20892/j.issn.2095-3941.2019.0268

**Published:** 2020-02-15

**Authors:** Peinan Chen, Xueke Zhao, Fuyou Zhou, Xin Song, Shoujia Hu, Yan Jin, Xianzeng Wang, Xuena Han, Zongmin Fan, Ran Wang, Bei Li, Wenli Han, Panpan Wang, Jilin Li, Lixin Wan, Liguo Zhang, Qide Bao, Fubao Chang, Yanru Qin, Zhiwei Chang, Jianwei Ku, Haijun Yang, Ling Yuan, Jingli Ren, Xuemin Li, Lidong Wang

**Affiliations:** ^1^State Key Laboratory of Esophageal Cancer Prevention & Treatment and Henan Key Laboratory for Esophageal Cancer Research of The First Affiliated Hospital, Zhengzhou University, Zhengzhou 450052, China; ^2^Department of Thoracic Surgery, Anyang Tumor Hospital, Anyang 455000, China; ^3^Department of Histology and Embryology, School of Basic Medical Sciences, Xinxiang Medical University, Xinxiang 453000, China; ^4^Department of Thoracic Surgery, Linzhou People’s Hospital, Linzhou 456550, China; ^5^Department of Pathology and Pathophysiology, School of Basic Medical Sciences, Zhengzhou University, Zhengzhou 450000, China; ^6^Department of Pathology, Linzhou Esophageal Cancer Hospital, Linzhou 456550, China; ^7^Department of Oncology, Nanyang Central Hospital, Nanyang 473000, China; ^8^Department of Thoracic Surgery, Xinxiang Central Hospital, Xinxiang 453000, China; ^9^Department of Oncology, Anyang District Hospital, Anyang 455000, China; ^10^Department of Thoracic Surgery, Linzhou Center Hospital, Linzhou 456550, China; ^11^Department of Oncology, the First Affiliated Hospital of Zhengzhou University, Zhengzhou 450000, China; ^12^Department of Gastroenterology, The Second Affiliated Hospital of Nanyang Medical College, Nanyang 473000, China; ^13^Department of Pathology, Anyang Tumor Hospital, Anyang 455000, China; ^14^Department of Radiotherapy, The Affiliated Cancer Hospital of Zhengzhou University (Henan Cancer Hospital), Zhengzhou 450000, China; ^15^Department of Pathology, The Second Affiliated Hospital of Zhengzhou University, Zhengzhou 450000, China; ^16^Department of Pathology, Hebei Provincial Cixian People’s Hospital, Cixian 056500, China

**Keywords:** Cervical esophageal cancer, survival, esophagectomy, radiochemotherapy

## Abstract

**Objective:** There are no comprehensive studies on survival outcomes and optimal treatment protocols for cervical esophageal cancer (CEC), due to its rare clinical prevalence. Our objective was to determine the relationship between pathological characteristics, treatment protocols, and survival outcomes in Chinese CEC patients.

**Methods:** A total of 500 Chinese CEC patients were selected from our 500,000 esophageal and gastric cardia carcinoma database (1973–2018). There were two main groups: patients treated with surgery, and patients receiving non-surgical treatments (radiotherapy, radiochemotherapy, and chemotherapy). The Chi-square test and Kaplan–Meier method were used to compare the continuous variables and survival.

**Results:** Among the 500 CEC patients, 278 (55.6%) were male, and the median age was 60.9 ± 9.4 years. A total of 496 patients (99.2%) were diagnosed with squamous cell carcinoma. In 171 (34.2%) patients who received surgery, 22 (12.9%) had undergone laryngectomy. In 322 (64.4%) patients who received non-surgical treatments, 245 (76.1%) received radiotherapy. Stratified survival analysis showed that only T stage was related with survival outcomes for CEC patients in the surgical group, and the outcomes between laryngectomy and non-laryngectomy patients were similar. It was noteworthy that the 5-year survival rate was similar in CEC patients among the different groups treated with surgery, radiotherapy, chemotherapy, or radiochemotherapy (*P* = 0.244).

**Conclusions:** The CEC patients had similar survival outcomes after curative esophagectomy and radiotherapy, including those with or without total laryngectomy. These findings suggest that radiotherapy could be the initial choice for treatment of Chinese CEC patients.

## Introduction

Cervical esophagus is defined as the short part of the esophagus between the lower border of the cricoid cartilage and the thoracic inlet (suprasternal notch), ~18 cm from the incisor teeth^1^. Studies from Western countries have indicated that cervical esophageal cancer (CEC) is rare in clinic prevalence, with a ratio of 2%–10% for all esophageal cancers^2–6^. Prior to this report, the study with the largest sample size was from Italy^5^. It included 363 CEC (10.5%) patients, out of 3,445 esophageal cancer patients^5^, but only 148 CEC patients were eligible for the final analysis. A study from Japan indicated that there were only 64 CEC patients reported within 22 years (1960–1982)^7^. In China, an accurate incidence for CEC is unknown, and there have been no reports with a larger sample size for CEC. The clinicopathological features and treatment outcomes of CEC patients in China are also largely unknown. After almost 30 years of effort, we have established a database of esophageal and gastric cardia cancers in China, including 410,000 esophageal cancers (97% esophageal squamous cell carcinomas and 1.5% primary esophageal adenocarcinomas) and 90,000 gastric cardia adenocarcinomas^8^. Among these patients, more than 200,000 cases have been successfully followed-up since 1973. The present study selected 500 CEC patients from this database. Our aim was to characterize the clinicopathological features and treatment outcomes for Chinese CEC patients. To the best of our knowledge, this Chinese study has the largest sample size of any CEC study.

## Materials and methods

### Patients and follow-up

All patients were from the 500,000 esophageal and gastric cardia carcinoma database (1973–2018), established by Henan Key Laboratory for Esophageal Cancer Research of The First Affiliated Hospital, Zhengzhou University^8^. To select the present study cohort, the database was reviewed. All patients with accurate tumor location records and treatments for esophageal cancer were considered for inclusion in this study (1973–2018). CEC was defined as the epicenter of the esophageal tumor found between the esophageal orifice and the sternal notch. All medical records were reviewed for consistency and completeness.

The follow-up was performed with patients with accurate addresses through either yearly telephone or home interviews, until the death of patients. The last follow-up was completed in December 2018. Of the 500 CEC patients, 466 patients (93.2%) were followed-up successfully.

### The collection of clinicopathological information and tumor staging

All clinical information for each patient was collected and digitalized based on in-patient medical records, including gender, age at diagnosis, family history (2 or more esophageal cancer patients in the same family within consecutive 3 generations), cigarette smoking, alcohol consumption, histopathology and treatment procedures. Pathological diagnosis was based on the medical record for each patient. All patients with esophagectomy were staged according to the 2002 American Joint Committee on Cancer (AJCC) tumor node metastasis staging system for esophageal cancer^9^.

### Treatments

In this study, based on the medical records, the treatments for all patients were classified as esophagectomy, radiotherapy, chemotherapy, and radiochemotherapy. Based on the procedure and approach, esophagectomy was classified as esophagectomy with or without laryngectomy, left or right thoracotomy, and transhiatal esophagectomy. The total radiation dose was generally 40–60 Gy with 2 Gy per fraction per day. The chemotherapy was usually performed using cisplatin with 5-fluorouracil or taxol. Of the 500 CEC cases, there were 171 cases treated with surgery, 245 cases treated with radiotherapy, 11 cases treated with chemotherapy, 66 cases treated with radiochemotherapy, and 7 cases without any medical treatment.

### Statistical analysis

Statistical analysis was performed using SPSS statistical software for Windows, version 21.0 (SPSS, Chicago. IL, USA). The *t*-test and Chi-square test were used to compare the differences of categorical and continuous variables between different CEC groups, respectively. The survival outcome was estimated by the Kaplan-Meier method and the multivariate Cox proportional hazards regression model. A value of *P <* 0.05 was considered statistically significant.

## Results

### Distribution of CEC patients by clinicopathological changes

**Table 1** shows the distribution of all CEC patients by gender, age, family history, cigarette smoking, alcohol consumption, histopathology and treatment procedures from 1973–2018. There were 278 males with a mean age of 60.6 ± 9.3 years and 222 females with a mean age of 61.3 ± 9.5 years. Family aggregation for CEC patients was evident with a family history in 29.0% of the patients. In addition, almost all female CEC patients had no history of cigarette smoking and alcohol consumption. In contrast, half of the male patients had a history of cigarette smoking (52.2%) and less than one-third of the male patients had alcohol consumption (24.6%). Nearly all of the CEC patients were diagnosed with squamous cell carcinoma (99.2%). There were only 3 CEC patients with primary esophageal adenocarcinoma (0.6%), and 1 case was mucoepidermoid carcinoma. Of the CEC patients, more than one-third received esophagectomy (34.2%), including 22 cases with laryngectomy (12.9%) and 90 cases with left thoracotomy (52.6%). Two-thirds of the CEC patients received non-surgical treatments (64.4%), in which 76.1% received radiotherapy.

### Pathological features of CEC patients with esophagectomy

The pathological features of 171 CEC patients with esophagectomy from 1973–2018 are summarized in **Table 2**, including differentiation, T and N status, pathological stages, and incisal edge residue. Most patients were at advanced stage (IIa + IIb + III *vs*. 0 + I, 87.7% *vs.* 12.3%) and better degeneration (G1+G2, 82.2%), and the percentage of positive incisal edge residue was much higher (14.0%).

### Comparison of perioperative parameters of CEC patients with esophagectomy between laryngectomy and non-laryngectomy procedures

**Table 3** shows the comparisons between laryngectomy and non-laryngectomy procedures by gender, age, differentiation, T and N status, pathological stage, incisal edge residue, and anastomotic leakage. It showed that the laryngectomy group had a significantly higher proportion of T3–T4 (90.9% *vs*. 45.0%, *P* = 0.000) and higher proportion of stage III (59.1% *vs.* 16.8%, *P* = 0.000), in other words, non-laryngectomy had lower percentage of earlier stage (0–II, 83.2% *vs.* 40.9%, *P* = 0.000). However, there were no difference between these 2 groups in the percentage of incisal edge residue (*P* = 0.699), and anastomotic leakage (*P* = 0.063).

### Treatment outcome analysis

In patients who had been followed-up successfully until December, 2018, 13.9% patients survived more than 10 years, with the longest survival time of 26.9 years. It was noteworthy that the 5-year survival rates were similar in CEC patients among the different groups treated with surgery, radiotherapy, chemotherapy, or radiochemotherapy (*P* = 0.244). In patients with or without radiotherapy, or radiochemotherapy before esophagectomy, the 5-year survival rates were similar (37.0% *vs*. 52.1%, *P* = 0.106).

### Stratification analysis of survival-related factors

**Figure 1:**
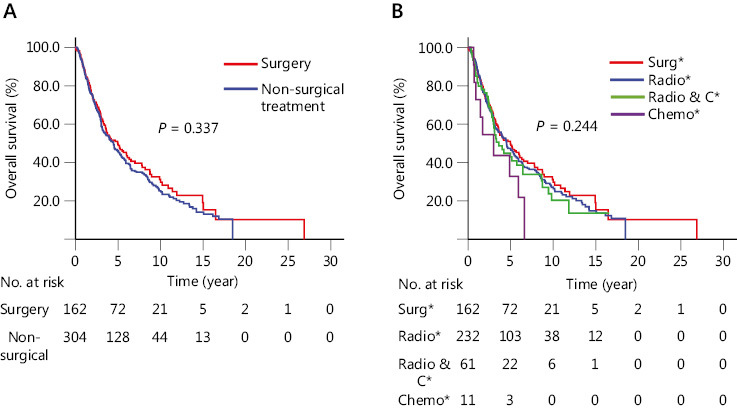
Kaplan–Meier curves comparing different treatment types of CEC patients. (A) Surgical and non-surgical treatments (i.e., radiotherapy, radiochemotherapy, and chemotherapy). (B) Different treatment types. ^*^Surg, curable surgery; Radio, radiotherapy; Radio & C, radiochemotherapy; Chemo, chemotherapy.

**Figure 2:**
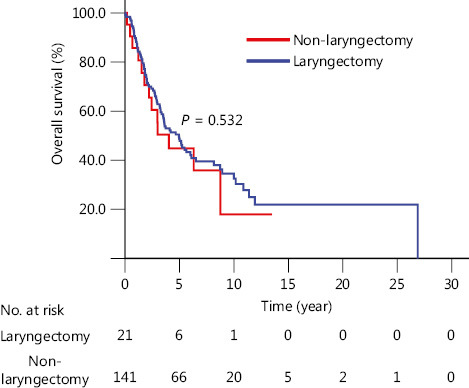
Kaplan-Meier curves comparing cervical esophageal cancer patients with or without laryngectomy.

For CEC patients, the 5-year survival rates were similar (*P* > 0.05) in terms of gender, age, family history, cigarette smoking, alcohol consumption, histological type (**Supplementary Figure S1A–F**), LNM (lymph node metastasis), and types of differentiation (**Supplementary Figure S2B, S2C**), either in CEC patients with esophagectomy with radiotherapy, chemotherapy, or radiochemotherapy (**Figure 1A, 1B**). In CEC patients with esophagectomy, although it seemed that patients with laryngectomy had a poorer 5-year survival rate, there was no significant difference (44.8% *vs*. 50.3%, *P* = 0.532) (**Figure 2**). In CEC patients, the tumor invasion depth was correlated with survival outcome. The 5-year survival rate in patients with T3 and T4 was lower than those with Tis (tumor *in situ*), T1, and T2 (37.7% *vs*. 61.7%, *P* = 0.026) (**Supplementary Figure S2A**). Although the CEC patients with negative LNM had a higher 5-year survival rate than those with positive LNM, the difference was not statistically significant (55.8% *vs*. 33.9%, *P* = 0.056) (**Supplementary Figure S2B**). There was no significant difference in the 5-year survival rate among different grades of differentiation for the CEC patients (*P* = 0.545) (**Supplementary Figure S2C**). Regarding pathological stage, CEC patients with early stage (0 and I) had significantly better survival rates than stage III patients (80.0% *vs*. 34.2%, *P* = 0.016) (**Supplementary Figure S2D**).

### Esophagectomy approach and survival analysis

In CEC patients, the 5-year survival rate was similar in patients with esophagectomy through left or right thoracotomy, and transhiatal approaches (49.8% *vs*. 47.3% *vs*. 50.1%, respectively, *P* = 0.696) (**Supplementary Figure S3B**).

### Treatment period analysis

We divided all CEC patients into 3 groups (1973–1997, 1998–2007, and 2008–2018) (**Supplementary Table S1**). The survival rates were similar between the surgical and non-surgical treatment groups in different time periods (64.3 *vs*. 68.8%, *P* = 0.912) (**Supplementary Figure S4A**); (48.1% *vs*. 51.3%, *P* = 0.922) (**Supplementary Figure S4B**); and (48.5% *vs.* 34.4%, *P* = 0.111) (**Supplementary Figure S4C**). For patients receiving radiotherapy, we combined the groups of 1973–1997 and 1998–2017 into 1 group. The ratio of CEC patients treated before 1997 was less than those in the other 2 time groups, and there was no significant difference in the 5-year survival rates (50.4% *vs*. 40.3%, *P* = 0.156) (**Supplementary Figure S5**).

## Discussion

To the best of our knowledge, this is the first report of Chinese CEC patients, with the largest sample size in terms of clinicopathological features and treatment outcomes. The present study showed a low CEC prevalence in the Chinese population. Moreover, CEC in China seemed to be more prevalent in females, predominantly involving squamous cell carcinomas.

In the present study, it was noteworthy that 3 CEC cases were identified as adenocarcinomas. Although adenocarcinoma is the major pathological type of esophageal cancer in Western countries, this phenomenon is difficult to explain using the Western model of esophageal adenocarcinoma formation, i.e., from reflux esophagitis to Barrett’s esophagus to dysplasia, and finally to an adenocarcinoma. Accumulated evidence has shown that primary esophageal adenocarcinomas in the Chinese population mostly originate from the esophageal propria gland or duct epithelium^10^. Endoscopic screening for high risk subjects and symptom-free early esophageal cancer patients in high incidence areas in Linxian, Henan showed a very low detection rate of Barrett’s esophagus and reflux esophagitis (as low as 0.7% and 4.5%, respectively)^11^. These findings suggest that different mechanisms in Chinese patients might be involved in the oncogenesis of CEC adenocarcinoma.

In our study, 29.0% of CEC patients had a positive family history. In China, family clustering has been observed for esophageal cancer in both low incidence and high incidence areas^12,13^. It has been reported that positive family history and genetic changes may increase the susceptibility to esophageal cancer,^14–16^ and esophageal cancer survival^17^. However, further studies are needed to further characterize the mechanism of family histories and the survival of CEC patients.

Regarding CEC survival outcomes, the present study showed that curative esophagectomy and non-surgical treatment as initial treatments for CEC had comparable survival outcomes (*P =* 0.337). For Chinese CEC patients, radiotherapy is usually the initial choice of most patient relatives or clinicians, because of the possible risk of laryngectomy or incisal edge residue due to complicated surgery. Reviewing recent reports^3,5,6,18,19^ on the comparisons between surgery and non-surgical treatments, we found there was no advantage of surgery (**Supplementary Table S2**).

The present study also showed that early CEC (Tis-1N0M0) with curative surgical treatment did not show any survival benefit when compared with advanced or progressed CEC, or with non-surgical treatment of CEC. Because of its rare prevalence, large series studies to compare different treatments of CEC in China are very limited. A few studies of CEC have shown that the survival outcomes for CEC are comparable for curative surgery and non-surgical treatment^3,5^. With improvements in radiotherapy and chemotherapy, the gold standard for CEC treatment has been changed from histologically pharyngo–laryngo–esophagectomy to adjuvant radiotherapy and/or chemotherapy, followed with or without surgery. Chen et al.^20^ reported that 63 CEC patients with concurrent radiochemotherapy had satisfactory outcomes.

Another interesting result in the present study was that in CEC patients, the 5-year survival rate was similar even in groups with esophagectomy plus laryngectomy or larynx preservation. There was no significant difference between the two different surgical procedures, although in the early 5-years or even longer times after the patients received esophagectomy with larynx preservation, they seemed to have better survival. Other group studies have recently reported that larynx-preserving esophagectomy for CEC is feasible and oncologically acceptable^21,22^. Our results also showed that patients receiving laryngectony did not have a lower rate of incisal edge residue. Considering the low quality of life following total laryngectomy and similar survival outcomes for esophagectomy and radiotherapy, or radiochemotherapy, the present study strongly suggested the advantage of radiotherapy as the initial choice in CEC treatment strategies. Our study showed that there was no difference in the survival rate of CEC patients who received radiotherapy between different decades. This might be because most cases enrolled in this study were from villages, so they were treated in local county hospitals in Henan province, which is an economically depressed area. It might be difficult to introduce new technology involving non-surgical treatments in these areas. Otherwise, in different decades, there was no advantage for surgical treatment, which suggests that CEC patients select radiotherapy or radiochemotherapy as the initial treatments.

The present results showed that the percentage of female patients with CEC was high, which was different from thoracic esophageal cancer. Saeki et al.^2^ also reported a higher percentage of female patients in the CEC group, and suggested that the higher percentage of female CEC patients might be due to cigarette smoking. Hoeben et al.^1^ reported that cigarette smoking and alcohol consumption were risk factors to CEC. Although consumptions of tobacco and alcohol have been recognized as risk factors for esophageal cancer, especially for esophageal squamous cell carcinoma^23^, in China, the historical and current consumptions of tobacco and alcohol in males are more common than in females. We therefore suggest that there might be other mechanisms involved in gender distributions in CEC patients, which needs to be further elucidated. A multidimensional analysis of molecular differences between male and female cancer patients has classified cancer types into two groups showing distinct incidence and mortality profiles. Extensive gender-biased gene expression signatures have been identified in some cancer types, which may be helpful in developing gender-speciﬁc therapeutic strategies and for elucidating the mechanisms involved in cancer related clinical controversy by gender^24^.

## Conclusions

The present study determined the clinicopathological characteristics of CEC patients in terms of gender, alcohol consumption, cigarette smoking, family history, LNM, anastomotic leakage, and incisal edge residues. In CEC patients, the survival outcomes with curative esophagectomy (with or without total laryngectomy) and radiotherapy were similar. Considering the low quality of life following total laryngectomy and anastomotic leakage, radiotherapy should be the initial choice for treatment of CEC in Chinese patients.

## Figures and Tables

**Table 1:** The distribution of 500 cases with CEC by clinicopathological information

Characteristics	CEC, *n* (%)
Gender	
Male	278 (55.6)
Female	222 (44.4)
Male/female	1/0.8
Mean age, years (SD)	60.9 (9.4)
Histological type	
Squamous cell carcinoma	496 (99.2)
Adenocarcinoma	3 (0.6)
Others	1 (0.2)
Family history^†^	
Positive	144 (29.0)
Negative	352 (71.0)
Cigarette smoking^‡^	
Yes	141 (28.8)
No	349 (71.2)
Alcohol consumption^§^	
Yes	66 (13.5)
No	424 (86.5)
Type of treatment	
Surgery	171 (34.2)
Radiotherapy	245 (49.0)
Radiochemotherapy	66 (13.2)
Chemotherapy	11 (2.2)
UR^*^	7 (1.4)

^*^CEC, cervical esophageal cancer; SD, standard deviation; UR, unrecorded patients for treatment procedures or out-patients.

^†^Four cases with missing family histories.

^‡^Ten cases with missing smoking information.

^§^Ten cases with missing alcohol consumption.

**Table 2:** The distributions of 171 CEC cases through esophagectomy according to clinicopathological information

Characteristics	CEC, *n* (%)
Gender	
Male	110 (64.3)
Female	61 (35.7)
Mean age, years, (SD)	58.9 ± 8.0
Histological type	
Squamous cell carcinoma	168 (98.2)
Adenocarcinoma	3 (1.8)
Others	0
T status	
Tis	3 (1.8)
T1	22 (12.9)
T2	59 (34.5)
T3	75 (43.8)
T4	12 (7.0)
N status	
pN (−)	125 (73.1)
pN (+)	46 (26.9)
Differentiation^†^	
High (G1)	27 (18.5)
Moderate (G2)	93 (63.7)
Low (G3)	26 (17.8)
Pathological stage	
0	3 (1.8)
I	18 (10.5)
IIa	101 (59.1)
IIb	11 (6.4)
III	38 (22.2)
IV	0
Preoperative treatment	
None	144 (84.2)
Chemotherapy	5 (2.9)
Radio ± C^*^	22 (12.9)
Surgical procedure	
Laryngectomy	22 (12.9)
Non-laryngectomy	149 (87.1)
Surgical approach	
Left thoracotomy	90 (52.6)
Right thoracotomy	24 (14.1)
Transhiatal esophagectomy	57 (33.3)
Incisal edge residue	
Negative	147 (86.0)
Positive	24 (14.0)

^*^CEC, cervical esophageal cancer; SD, standard deviation; Radio ± C, radiotherapy with or without chemotherapy.

^†^Twenty-five cases missing differentiation information.

**Table 3:** The comparison of 171 CEC cases through esophagectomy with or without laryngectomy according to clinicopathological information

Characteristics	Non-laryngectomy, *n* (%)	Laryngectomy, *n* (%)	*P*
Gender			0.005
Male	90 (60.4)	20 (90.9)	
Female	59 (39.6)	2 (9.1)	
Age			0.200
≥ 60	73 (49)	14 (63.6)	
< 60	76 (51)	8 (36.4)	
T status			0.000
Tis-T2	82 (55)	2 (9.1)	
T3-T4	67 (45)	20 (90.9)	
N status			0.036
N0	113 (75.8)	12 (54.5)	
N1	36 (24.2)	10 (45.5)	
G status^†^			0.093
G1-2	107 (84.3)	13 (68.4)	
G3	20 (15.7)	6 (31.6)	
Pathological stage			0.000
0–II	124 (83.2)	9 (40.9)	
III–IV	25 (16.8)	13 (59.1)	
Incisal edge residue			0.699
Negative	127 (85.2)	20 (90.9)	
Positive	22 (14.8)	2 (9.1)	
Anastomotic Leakage			0.063
Negative	122 (81.9)	22 (100)	
Positive	27 (18.1)	0	

^†^Twenty-five cases missing differentiation information.

## References

[1] Hoeben A, Polak J, Van De Voorde L, Hoebers F, Grabsch HI, de Vos-Geelen J (2016). Cervical esophageal cancer: a gap in cancer knowledge. Ann Oncol.

[2] Saeki H, Tsutsumi S, Yukaya T, Tajiri H, Tsutsumi R, Nishimura S (2017). Clinicopathological features of cervical esophageal cancer: retrospective analysis of 63 consecutive patients who underwent surgical resection. Ann Surg.

[3] Takebayashi K, Tsubosa Y, Matsuda S, Kawamorita K, Niihara M, Tsushima T (2017). Comparison of curative surgery and definitive chemoradiotherapy as initial treatment for patients with cervical esophageal cancer. Dis Esophagus.

[4] Li HX, Liu J, Cheng Y, Liu MN, Fang WT, Lv CX (2018). Concurrent chemoradiotherapy for cervical esophageal squamous cell carcinoma: treatment results from a prospective observational study. Dis Esophagus.

[5] Valmasoni M, Pierobon ES, Zanchettin G, Briscolini D, Moletta L, Ruol A (2018). Cervical esophageal cancer treatment strategies: a cohort study appraising the debated role of surgery. Ann Surg Oncol.

[6] Grass GD, Cooper SL, Armeson K, Garrett-Mayer E, Sharma A (2015). Cervical esophageal cancer: a population-based study. Head Neck.

[7] Kakegawa T, Yamana H, Ando N (1985). Analysis of surgical treatment for carcinoma situated in the cervical esophagus. Surgery.

[8] Wang XM, Gao HJ (2017). Exploration and practice in clinical biobanks.

[9] Greene FL, Page DL, Fleming ID, Fritz A, Balch CM, Haller DG (2002). AJCC Cancer Staging Manual (6th Edition).

[10] Zhang YX, Qiao SJ, Shen Q, Qiu SL, Song MQ, Yan AH (1994). Study on histology and pathology of primary esophageal adenocarcinoma in high incidence region of esophageal cancer in China. Henan Med Res.

[11] Wang LD, Zheng S, Zheng ZY, Casson AG (2003). Primary adenocarcinomas of lower esophagus, esophagogastric junction and gastric cardia: in special reference to China. World J Gastroenterol.

[12] Yang CS (1980). Research on esophageal cancer in China: a review. Cancer Res.

[13] Gao Y, Hu N, Han X, Giffen C, Ding T, Goldstein A (2009). Family history of cancer and risk for esophageal and gastric cancer in Shanxi, China. BMC Cancer.

[14] Hu N, Wang Z, Song X, Wei L, Kim BS, Freedman ND (2016). Genome-wide association study of gastric adenocarcinoma in Asia: a comparison of associations between cardia and non-cardia tumours. Gut.

[15] Wang LD, Zhou FY, Li XM, Sun LD, Song X, Jin Y (2010). Genome-wide association study of esophageal squamous cell carcinoma in Chinese subjects identifies susceptibility loci at PLCE1 and C20orf54. Nat Genet.

[16] Wu C, Wang Z, Song X, Feng XS, Abnet CC, He J (2014). Joint analysis of three genome-wide association studies of esophageal squamous cell carcinoma in Chinese populations. Nat Genet.

[17] Wu C, Li D, Jia W, Hu Z, Zhou Y, Yu D (2013). Genome-wide association study identifies common variants in SLC39A6 associated with length of survival in esophageal squamous-cell carcinoma. Nat Genet.

[18] Tong DK, Law S, Kwong DL, Wei W I, Ng RW, Wong KH (2011). Current management of cervical esophageal cancer. World J Surg.

[19] Cao CN, Luo JW, Gao L, Xu GZ, Yi JL, Huang XD (2014). Primary radiotherapy compared with primary surgery in cervical esophageal cancer. JAMA Otolaryngol Head Neck Surg.

[20] Chen YH, Lu HI, Lo CM, Wang YM, Chou SY, Hsiao CC (2019). The clinical outcomes of locally advanced cervical esophageal squamous cell carcinoma patients receiving curative concurrent chemoradiotherapy: a population-based propensity score-matched analysis. Cancers (Basel).

[21] Sun F, Li X, Lei D, Jin T, Liu D, Zhao H (2014). Surgical management of cervical esophageal carcinoma with larynx preservation and reconstruction. Int J Clin Exp Med.

[22] Makino T, Yamasaki M, Miyazaki Y, Takahashi T, Kurokawa Y, Takiguchi S (2016). Short- and long-term outcomes of larynx-preserving surgery for cervical esophageal cancer: analysis of 100 consecutive cases. Ann Surg Oncol.

[23] Rustgi AK, El-Serag HB (2014). Esophageal carcinoma. N Engl J Med.

[24] Yuan Y, Liu L, Chen H, Wang Y, Xu Y, Mao H (2016). Comprehensive characterization of molecular differences in cancer between male and female patients. Cancer Cell.

