# Supplementary material for: Characterization of 500 Chinese patients with cervical esophageal cancer by clinicopathological and treatment outcomes
Source: Cancer Biol Med. 2020 Feb 15;17(1):218–26. doi: 10.20892/j.issn.2095-3941.2019.0268 (PMC7142830; doi:10.20892/j.issn.2095-3941.2019.0268)
Supplement: Supplementary file 1 [file cbm-17-218-s001.pdf]

Supplementary materials

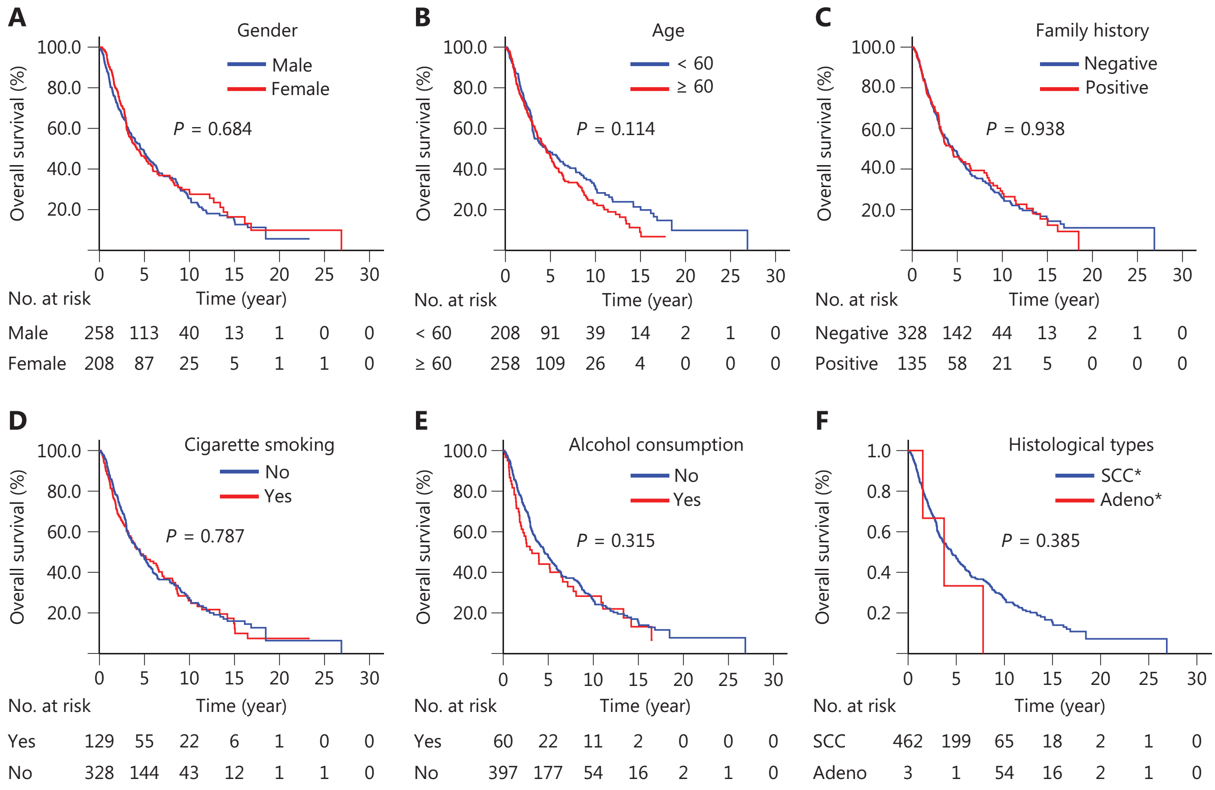

Figure S1 Kaplan–Meier curves comparing different gender, age, family history, cigarette smoking, alcohol consumption, and histological type in cervical esophageal cancer patients. Male and female (A); patients < 60 and ≥ 60 years of age (B); with and without a positive family history (C); with and without cigarette smoking (D); with and without alcohol consumption (E); and different histological types (F).

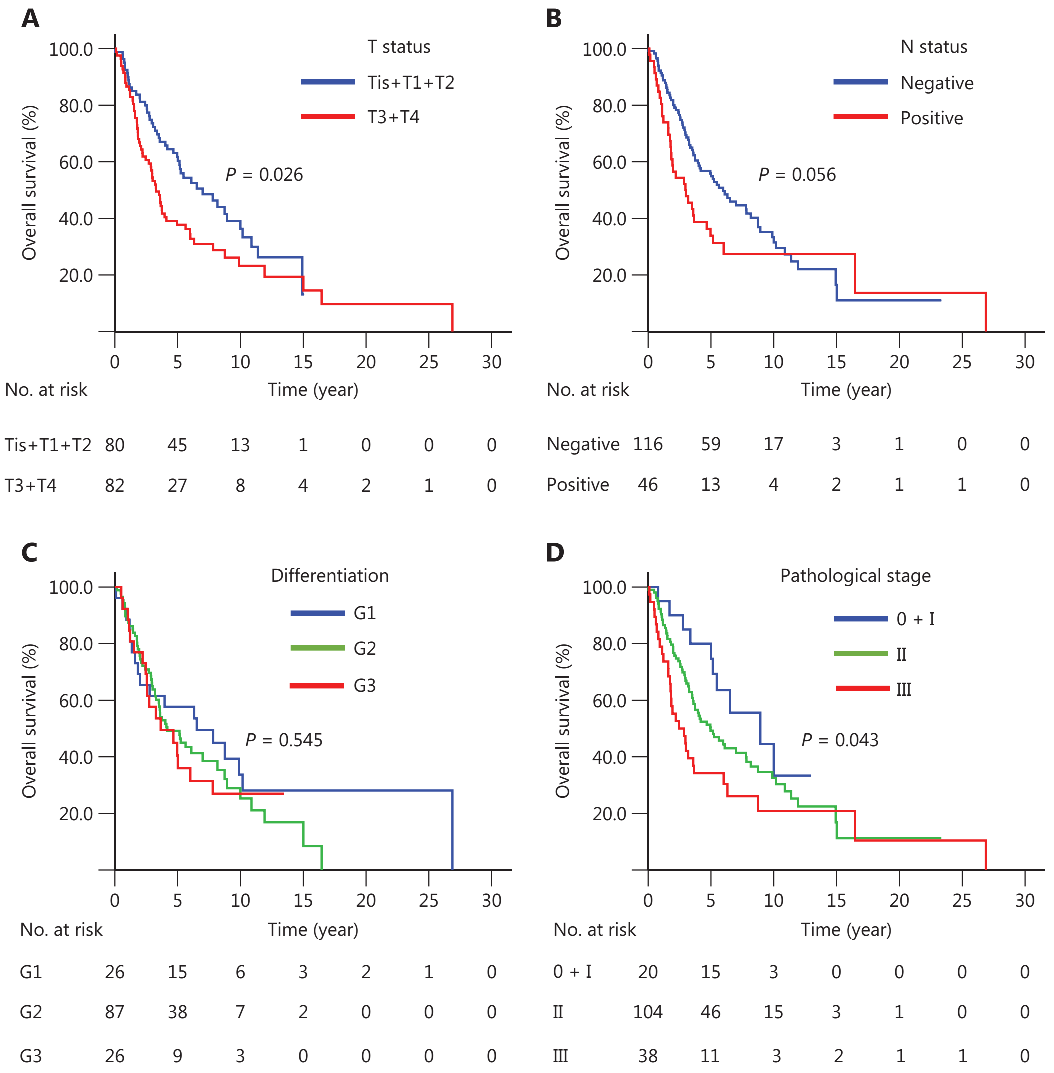

Figure S2 Kaplan–Meier curves comparing different T and N status, differentiation, and pathological stage in cervical esophageal cancer patients. Different T status (A); different N status (B); different differentiation (C); and different pathological stage (D).

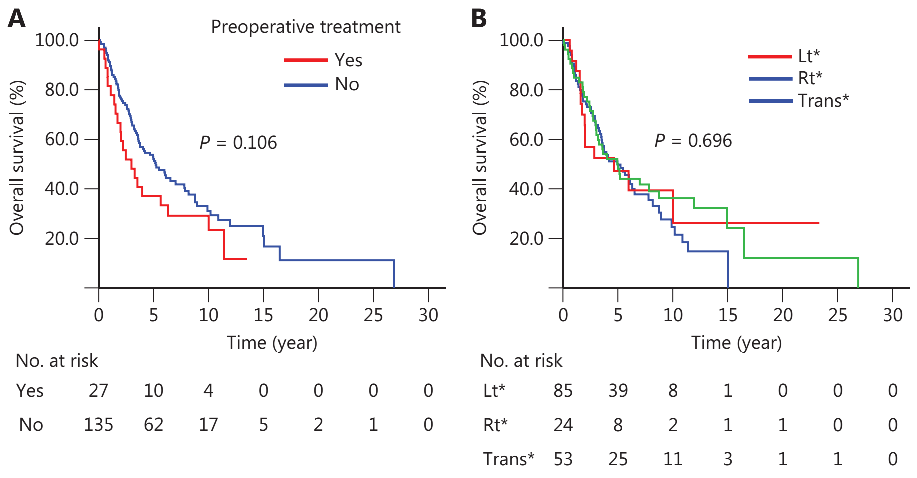

Figure S3 Kaplan–Meier curves comparing with and without preoperative treatment (A) and different surgical approaches (B) in cervical esophageal cancer patients.

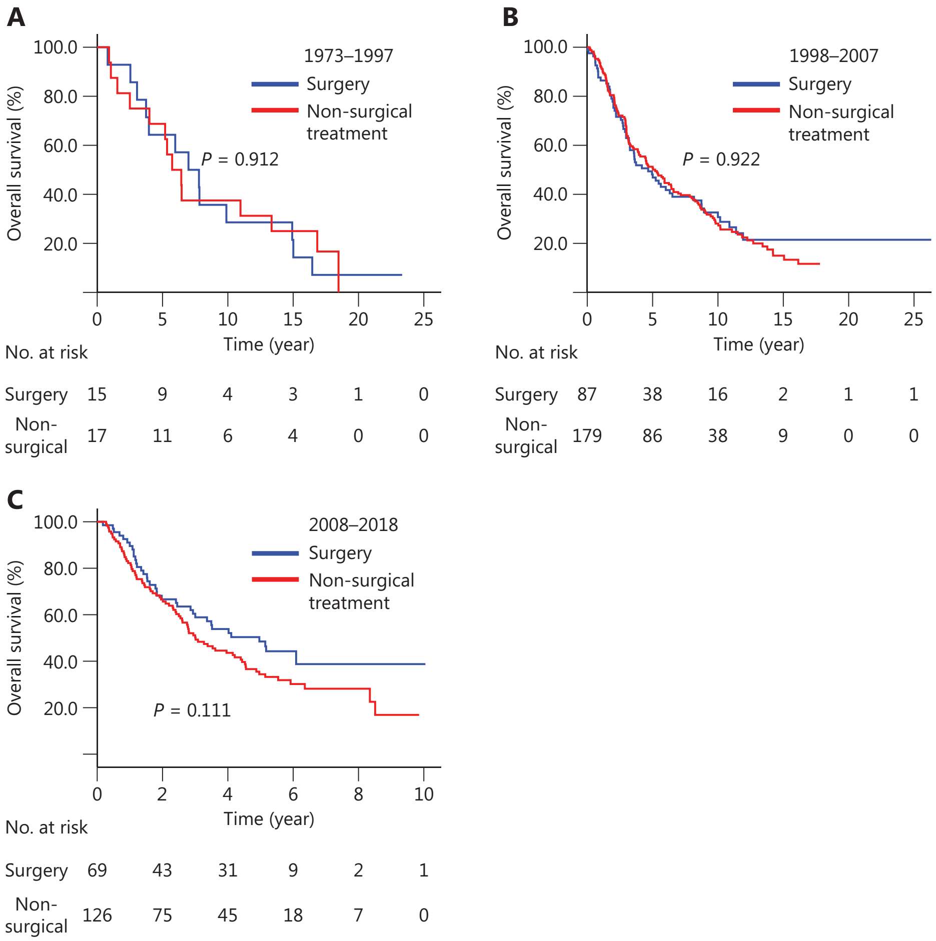

Figure S4 Kaplan–Meier curves comparing surgical and non-surgical treatment of cervical esophageal cancer patients in different periods: 1973–1997 (A); 1998–2007 (B); and 2008–2018 (C).

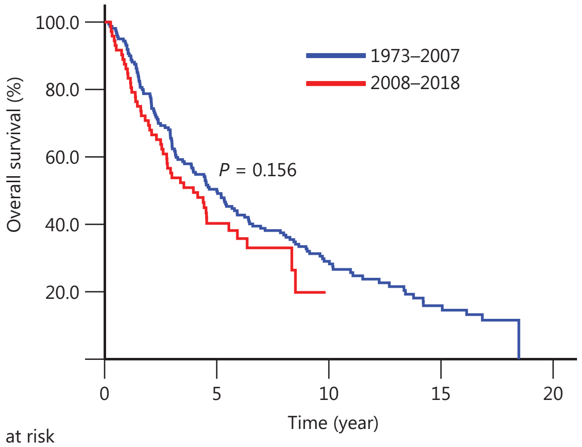

No. at risk

1973–2007	168	80	38	12	0
2008–2018	77	23	–	–	–

Figure S5 Kaplan–Meier curves comparing cervical esophageal cancer patients receiving radiotherapy during different periods (1973–2007 *vs*. 2008–2018).

Table S1 The distributions of 500 cases with CEC by treatment type during different periods

Period	*n* (%)				
	Surgery	Radiotherapy	Radiochemotherapy	Chemotherapy	UR
1973–1997	15 (8.8)	11 (4.5)	5 (7.6)	1 (9.1)	0
1998–2007	87 (50.9)	157 (64.1)	19 (28.8)	3 (27.3)	4 (57.1)
2008–2018	69 (40.3)	77 (31.4)	42 (63.6)	7 (63.6)	3 (42.9)

CEC: cervical esophageal cancer; UR, unrecorded patients for treatment procedure information or out-patients.

Table S2 Literature comparison between surgical and non-surgical treatments of CEC

Author	Country/region	Treatment	*n*	2 y	3 y	5 y	*P*
Takebayashi K[3]	Japan	Surgery	13			60.6	0.89
		CRT	36			51.4	
Valmasoni M[5]	Italy	surgery	56			12.6	0.088
		CRT	52			26.7	
		CRT+surgery	40			30.7	
Grass GD[6]	USA	Surgery	32		48.0	43.0	> 0.05
		CRT	240		33.0	28.0	
Tong DKH[18]	Hong Kong, China	CTRT	21	46.9			0.39
		Surgery	62	37.6			
Cao CN[19]	China	Surgery	63	50.7			0.31
		RT	161	49.3			
Chen PN, current study	China	Surgery	171	73.3	63.9	49.6	0.337
		Non-surgical	322	75.1	60.5	46.0	

CEC, cervical esophageal cancer; y, year; CRT, chemoradiotherapy; CTRT, chemoradiation; RT, radiotherapy.
